# An examination of data from the American Gut Project reveals that the dominance of the genus *Bifidobacterium* is associated with the diversity and robustness of the gut microbiota

**DOI:** 10.1002/mbo3.939

**Published:** 2019-09-30

**Authors:** Yuqing Feng, Yunfeng Duan, Zhenjiang Xu, Na Lyu, Fei Liu, Shihao Liang, Baoli Zhu

**Affiliations:** ^1^ CAS Key Laboratory of Pathogenic Microbiology and Immunology Institute of Microbiology Chinese Academy of Sciences Beijing China; ^2^ Savaid Medical School University of Chinese Academy of Sciences Beijing China; ^3^ School of Food Science & Technology Nanchang University Nanchang China; ^4^ Beijing Key Laboratory of Antimicrobial Resistance and Pathogen Genomics Beijing China; ^5^ Department of Pathogenic Biology School of Basic Medical Sciences Southwest Medical University Luzhou China

**Keywords:** American Gut Project, *Bifidobacterium*, diversity, *Lactobacillus*, network, zero‐inflated negative binomial

## Abstract

*Bifidobacterium* and *Lactobacillus* are beneficial for human health, and many strains of these two genera are widely used as probiotics. We used two large datasets published by the American Gut Project (AGP) and a gut metagenomic dataset (NBT) to analyze the relationship between these two genera and the community structure of the gut microbiota. The meta‐analysis showed that *Bifidobacterium*, but not *Lactobacillus*, is among the dominant genera in the human gut microbiota. The relative abundance of *Bifidobacterium* was elevated when *Lactobacillus* was present. Moreover, these two genera showed a positive correlation with some butyrate producers among the dominant genera, and both were associated with alpha diversity, beta diversity, and the robustness of the gut microbiota. Additionally, samples harboring *Bifidobacterium* present but no *Lactobacillus* showed higher alpha diversity and were more robust than those only carrying *Lactobacillus*. Further comparisons with other genera validated the important role of *Bifidobacterium* in the gut microbiota robustness. Multivariate analysis of 11,744 samples from the AGP dataset suggested *Bifidobacterium* to be associated with demographic features, lifestyle, and disease. In summary, *Bifidobacterium* members, which are promoted by dairy and whole‐grain consumption, are more important than *Lactobacillus* in maintaining the diversity and robustness of the gut microbiota.

## Graphical abstract

Our study has shown that Bifidobacterium, as a dominant genus, played an important role in terms of the diversity and stability of gut microbiota examined using American Gut Project. It is more helpful to increase the alpha diversity and the stability of gut microbiota than *Lactobacillus*. Furthermore, we found that whole‐grain consumption and fruit consumption could increase the abundance of *Bifidobacterium*.

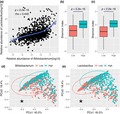

## 1 INTRODUCTION

The human gut is colonized by an abundance of bacteria, with an estimated count of 3 × 10^13^ (Sender, Fuchs, & Milo, 2016). The human gut is normally colonized by three groups of bacteria: commensals, pathobionts, and probiotics (Vitetta, Saltzman, Nikov, Ibrahim, & Hall, 2016). The bacterial species most often utilized as probiotics are from the genera *Bifidobacterium* and *Lactobacillus*, which are proven to be beneficial to human health (Salminen et al., 1998). Various strains of *Bifidobacterium* and *Lactobacillus* have been reported to suppress diarrhea, alleviate lactose intolerance and postoperative complications, exhibit antimicrobial and anticolorectal cancer activities, reduce symptoms of irritable bowel syndrome (IBS), and prevent inflammatory bowel disease (IBD) (Bermudez‐Brito, Plaza‐Díaz, Muñoz‐Quezada, Gómez‐Llorente, & Gil, 2012). The diversity and robustness of the bacterial community in any ecosystem are two aspects usually explored in ecological studies (Ives & Carpenter, 2007), and greater diversity of the intestinal microbiota appears to be associated with better health (Claesson et al., 2012). However, the conclusions of previous studies regarding whether oral administration of *Bifidobacterium* and *Lactobacillus* species increases the alpha diversity of the human gut microbiota are not consistent (Karlsson et al., 2010; Kato‐Kataoka et al., 2016; van Zanten et al., 2014). In addition, the role played by *Bifidobacterium* and *Lactobacillus* in diseases, such as IBS (Cozmapetruţ, Loghin, Miere, & Dumitraşcu, 2017), and allergy (Mennini, Dahdah, Artesani, Fiocchi, & Martelli, 2017) remains uncertain. Apart from the facts mentioned above, most previous studies focus on the diversity, community composition and their variation of the gut microbiota, and rarely on the relationships between microbial species (Li & Wu, 2018). At the same time, bacterial network analysis gives new insight into the interspecies interaction of bacterial communities and promotes the understanding of the niche spaces among community members (Barberán, Bates, Casamayor, & Fierer, 2012). To our knowledge, the effect of certain taxa on the bacterial network has rarely been reported. To build a bacterial network, it will be difficult to determine whether or not cooccurrence patterns are statistically significant without a sufficiently large sample set (Barberán et al., 2012).

However, only a few large datasets for the gut microbiota have been constructed. To our knowledge, the American Gut Project (AGP) is one of the largest datasets on the human gut microbiota (http://americangut.org/about/). Regardless, the return of samples through the mail at room temperature without preservatives, possibly leading to the outgrowth of some bacteria in the samples, is a limitation of the AGP dataset (http://americangut.org/how-it-works/). It should be noted that researchers of the AGP group proved the feasibility of correcting the microbiome profiles in the AGP dataset by deleting “blooming” taxa to ensure that the results obtained from the dataset are trustworthy (Amir et al., 2017). The gut metagenome dataset published by Li et al., (2014) (NBT) is another large dataset, consisting of 1,267 fecal samples. The number of samples in these gut metagenome datasets is large enough for use in further validation.

Although many studies have focused on characterizing the function of these two genera, there are very few studies about the correlation between them and the community structure of the bacterial network. Therefore, we designed the present study to analyze the relationship between these two genera and the community structure of the gut microbiota to explore the potential role of these two genera to the characterizations of the gut microbiota.

## 2 METHODS

### 2.1 Data acquisition and processing

**Figure A1:**
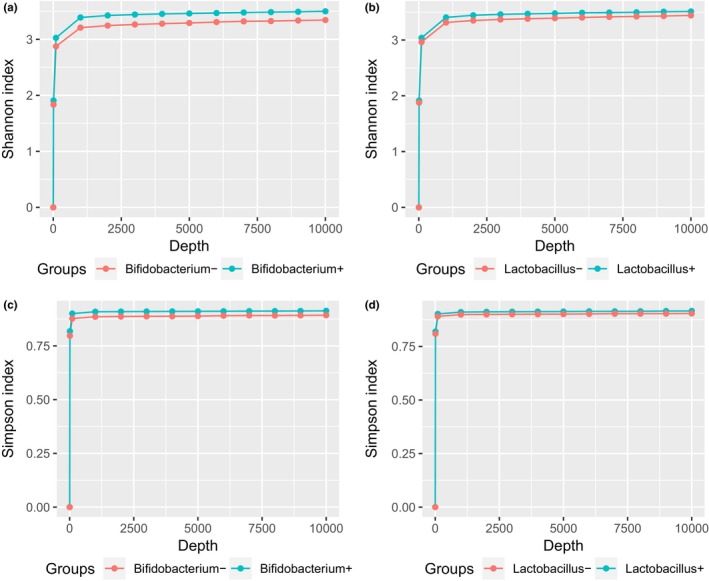
Rarefaction curves of alpha diversity. Rarefaction curves on Shannon index (a,b), and Simpson index (c,d)

Construction of the AGP dataset was accompanied by the completion of metadata questionnaires, which included questions on demographic features, lifestyle, and disease. To avoid bias caused by DNA extraction, library preparation methods, and the sequencing platform (Costea et al., 2017), all samples were analyzed via the procedure described in the Earth Microbiome Project (Earth Microbiome Project 16S Illumina Amplicon Protocol, http://press.igsb.anl.gov/earthmicrobiome/protocols-and-standards/16s/). Raw data and information from the questionnaires were downloaded from EBI (Accession #ERP012803). DADA2 was used to infer the amplicon sequence variants (ASVs) present in each sample (Callahan et al., 2016). Forward reads were trimmed and filtered, with reads truncated at 140 nt, no ambiguous bases allowed, and each read required to have less than two expected errors based on quality scores. Taxonomic assignment was performed against the Silva v132 database (Quast et al., 2012). We performed species‐level assignments based on exact matching by using addSpecies in DADA2. To avoid bias caused by the sequencing depth, we collected sequencing data for fecal samples with one criterion: More than ten thousand sequencing reads must be available for each sample (Figure A1 in Appendix 1). We selected 12,127 gut samples (AGP dataset) from the dataset of 19,327 samples (downloaded on Jan. 25, 2018). Due to the low quality of some sequencing data, we excluded 383 samples from the cohort. Furthermore, we deleted the top 10 “blooming” taxa suggested by Amir and colleagues to yield results consistent with published microbiome studies performed using frozen or otherwise preserved samples (Amir et al., 2017). To simplify downstream analysis, we applied a frequency filter for 128,145 ASVs, where taxa were retained only if they were found in at least 1% of the samples (117 samples), according to a previous study (Fitzpatrick et al., 2018). Ultimately, we obtained a dataset consisting of 11,744 samples with 1,409 ASVs, with 8,629 samples from the USA and 2,560 from the United Kingdom; the majority of the individuals represented in the dataset are Caucasian White (*n* = 10,201) (Table A1 in Appendix 1). Considering that the sample from the AGP dataset is very heterogeneous with many diseases, we excluded samples from infants and individuals with diseases (Table A2 in Appendix 1), which might cause bias in the further analysis (Stewart et al., 2018; Tremaroli & Backhed, 2012). Finally, 2,186 samples were included in the ensuing analysis (Table A3 in Appendix 1).

To further test the results obtained from the AGP dataset, we downloaded a genus profile for 1,267 samples (http://meta.genomics.cn/meta/dataTools). These data were generated from high‐throughput metagenomic sequencing and annotated based on reference genomes to obtain the relative abundance of the genera in the profile (Li et al., 2014). This dataset consisted of 760 European samples (Le Chatelier et al., 2013; Nielsen et al., 2014; Qin et al., 2010), 368 Chinese samples (Qin et al., 2012), and 139 American samples (Methe et al., 2012).

### 2.2 Identification of dominant genera

A previous study first proposed the concept of dominant soil bacterial phylotypes, which represents a small subset of phylotypes that account for almost half of the 16S rRNA sequences recovered from soils, allowing the prediction of how future environmental change will affect the spatial distribution of these taxa (Delgado‐baquerizo et al., 2018). In our analysis of AGP data, we introduced the concept of dominant genera, which include those that are highly abundant (the top 10% most frequently found genera sorted by their percentage of relative abundance) and ubiquitous (found in more than 70% of the samples evaluated) (Delgado‐baquerizo et al., 2018; Soliveres et al., 2016).

### 2.3 Distance analysis of ASVs annotated as *Bifidobacterium* and *Lactobacillus*

Complete 16S rRNA gene sequences of species belonging to *Bifidobacterium* and *Lactobacillus* were downloaded from the SILVA database (Quast et al., 2012). Distance trees were constructed based on sequences of the V4 region via a neighbor‐joining algorithm (with 500 bootstrap replicates) available in Mega 7 software (Kumar, Stecher, & Tamura, 2016). Representative sequences from each species were randomly selected.

### 2.4 Diversity analysis

Alpha diversity was calculated using the vegan package (Zapala & Schork, 2006) in R software. Six indexes were applied in the analysis: the Shannon index, Chao1 index, observed ASVs, ACE index, inverse Simpson index, and Pielou index. Principal coordinate analysis (PCoA) was conducted using the data of Bray–Curtis dissimilarity data (Bray & Curtis, 1957). To assess whether the presence of the two genera was a significant factor for explaining variation in the gut microbiota, we devided the continuous variables of their abundance into categorical variables as explanatory factors. Taking *Bifidobacterium,* for example, we introduced two categories as explanatory factors according to its presence or not: One category is the samples with *Bifidobacterium* and the other is the samples without *Bifidobacterium*. And, permutational multivariate analysis of variance (PERMANOVA) was applied with a parameter of 9,999 permutations in R (Zapala & Schork, 2006).

### 2.5 Construction of microbial networks

Microbial network analysis has been employed to examine keystone taxa and relationships among the microbial community, which can provide useful information for further intervention (Banerjee, Schlaeppi, & van der Heijden, 2018). In the present study, we applied SParse InversE Covariance Estimation for Ecological ASsociation Inference (SPIEC‐EASI), a statistical method for the inference of microbial ecological networks from amplicon sequencing datasets (Kurtz et al., 2015). The network was constructed based on relative abundance at the genus level following the instructions at https://github.com/zdk123/SpiecEasi. Considering that increasing the rep.num argument may result in better performance (Kurtz et al., 2015), networks were constructed using the SPIEC‐EASI package in R with the default parameters, except that the parameters nlambda and rep.num were each set as 100 (Liu et al., 2017). The degree statistics is a measure of the centrality of nodes, with higher values indicating that the node is involved in more ecological interactions. We assessed the robustness of the different microbial association networks to random node removal (“attack”) (Albert, Jeong, & Barabasi, 2000; Iyer, Killingback, Sundaram, & Wang, 2013) using natural connectivity (Jun, Barahona, Yue‐Jin, & Hong‐Zhong, 2010) as a general measure of graph stability. We also measured how the natural connectivity of the microbial network changed when nodes and their associated edges were removed from the network (Mahana et al., 2016).

### 2.6 Regression analysis

Because of excessive zero abundance in the read counts and the overdispersion, a multiple zero‐inflated negative binomial (ZINB) regression model (Alan, 2015) was used to determine the differential abundance in the analysis of *Bifidobacterium*. The ZINB model consists of two different components: A logistic regression component for modeling excessive zeros and a negative binomial regression component for modeling the remaining count values. Missing data in each categorical variable were included in a separate hidden category (Hill, 2006). Overall, fitted mean proportions were calculated by the average predicted value (APV) method (Albert, Wang, & Nelson, 2014), in which *Bifidobacterium* count values are divided by the mean total read counts under each exposure status. The variables of host features were selected based on the record number and biological relevance, and 16 variables were retained for further study, namely, age, sex, race, geographical location, whole‐grain consumption, vegetable consumption, fruit consumption, milk and cheese consumption, C‐section, feeding patterns, antibiotic exposure, IBD, IBS, autoimmune disease, cardiovascular disease, and food allergy. To allow clear interpretation of the result, we divided frequency into three categories, “high frequency,” “low frequency,” and “never”. We divided the race into five categories, namely, “Caucasian White” (CW), “African‐American” (AA), “Hispanic” (HI), “Asian‐Pacific” (AP), and “Other”. We also divided geographical location into four new categories, namely, “North American” (NA), “Europe” (EU), “Oceania” (OC), and “Other”.

### 2.7 Statistical analysis

Statistical significance of the overlap was performed online (http://nemates.org/MA/progs/overlap_stats.html) and chi‐square test. Differences between groups were tested using Wilcoxon rank‐sum test. When multiple hypotheses were considered simultaneously, p‐values were adjusted to control the false discovery rate with the method described previously (Benjamini & Hochberg, 1995).

## 3 RESULTS

### 3.1 *Bifidobacterium* is a dominant genus in the human gut microbiota

**Figure 1:**
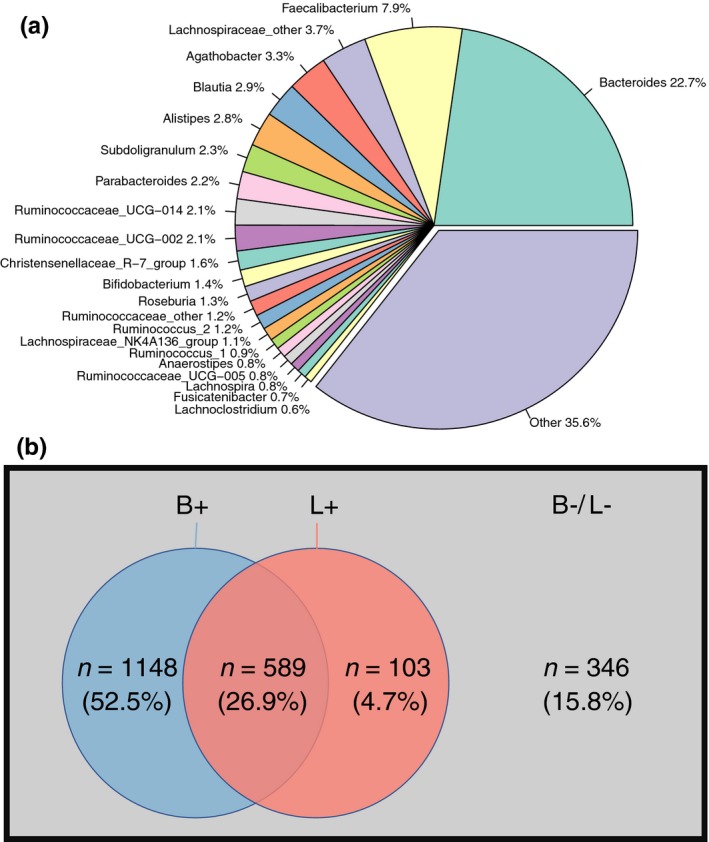
Composition and distribution of genera in the AGP dataset. (a) Genus composition among the 2,520 fecal samples in the AGP dataset. (b) Euler diagram of the cooccurrences of *Bifidobacterium* and *Lactobacillus* in samples. B+: samples containing *Bifidobacterium*; L+: samples containing *Lactobacillus*; B‐/L‐: samples containing neither *Bifidobacterium* nor *Lactobacillus*

Based on the criteria for defining dominant genera outlined in the Methods section, only 8.0% (22/276) of the bacterial genera among the 2,186 samples were dominant. However, this small number of genera accounted for an average of 64.4% of the relative abundance (Figure 1a). *Bifidobacterium* was among the dominant genera, whereas *Lactobacillus* was not subsamples from the USA and UK also showed that *Bifidobacterium*, but not *Lactobacillus*, was a dominant genus (Table A4, A5, A6 in Appendix 1). The significance of the overlap test suggested that the distribution of these two genera exhibited a close connection (Figure 1b, *p* < .001, chi‐square test). We also validated the result using another online statistic service (http://nemates.org/MA/progs/overlap_stats.html), and the result also revealed a close connection between *Bifidobacterium* and *Lactobacillus* (*p* < 3.6 × 10^−6^).

**Figure A2:**
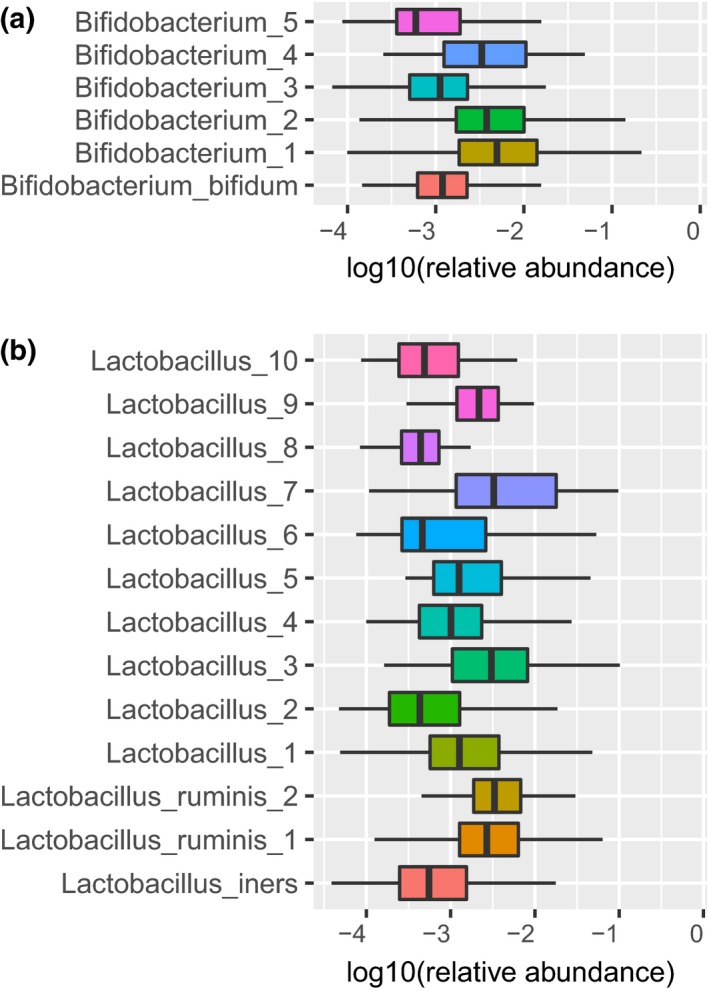
Relative abundance of ASVs annotated as (a) *Bifidobacterium* and (b) *Lactobacillus*

**Figure A3:**
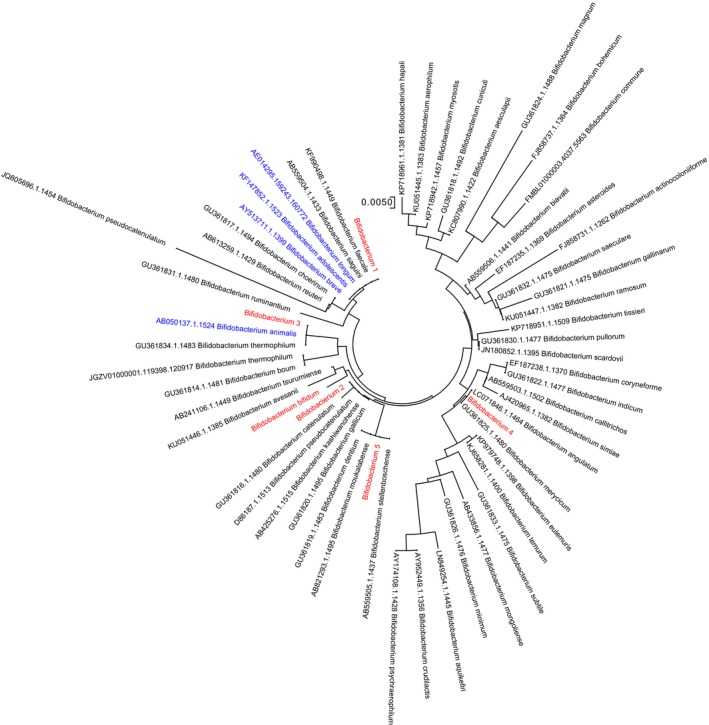
Distance tree of the genus *Bifidobacterium*. The red branches denote ASVs annotated as *Bifidobacterium*; the blue branches denote species commonly used as probiotics

**Figure A4:**
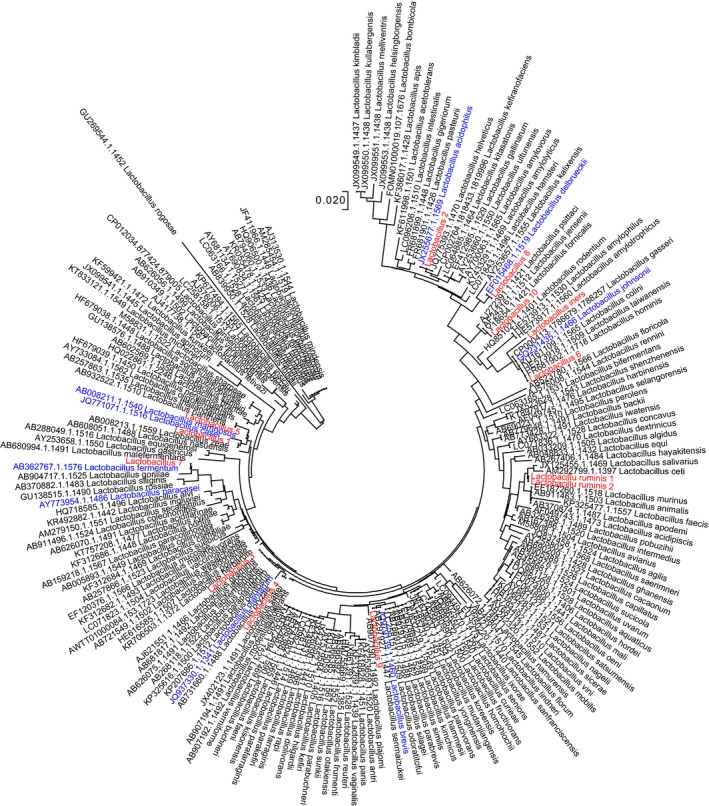
Distance tree of the genus *Lactobacillus*. The red branches denote ASVs annotated as *Lactobacillus*; the blue branches denote species commonly used as probiotics

Among the remaining 1,409 ASVs, 6 and 13 ASVs were annotated as *Bifidobacterium* and *Lactobacillus*, respectively (Table A7 in Appendix 1). The relative abundance of each ASV annotated as *Bifidobacterium* or *Lactobacillus* varied significantly, with only some ASVs dominating each genus (Figure A2). Although with the limitation of amplicon length makes it difficult to classify ASVs at the species level (Figure A3 and Figure A4), we still found that some ASVs showed high identity (98.6%–100.0%) to species commonly used as probiotics, namely, *Bifidobacterium*_1 (*Bifidobacterium longum*, *Bifidobacterium adolescentis,* and *Bifidobacterium breve*)*, Bifidobacterium*_3 (*Bifidobacterium animalis*)*, Lactobacillus*_1 (*Lactobacillus casei*)*, Lactobacillus*_2 (*Lactobacillus acidophilus*)*, Lactobacillus*_5 (*Lactobacillus rhamnosus*)*, Lactobaicllus*_7 (*Lactobacillus fermentum*)*, Lactobaicllus*_8 (*Lactobacillus delbrueckii*)*, and Lactobacillus*_9 (*Lactobacillus brevis*)*.* These ASVs also exhibited high relative abundance for *Bifidobacterium* and *Lactobacillus*.

### 3.2 *Bifidobacterium* and *Lactobacillus* are associated with the diversity of the gut microbiota

**Figure 2:**
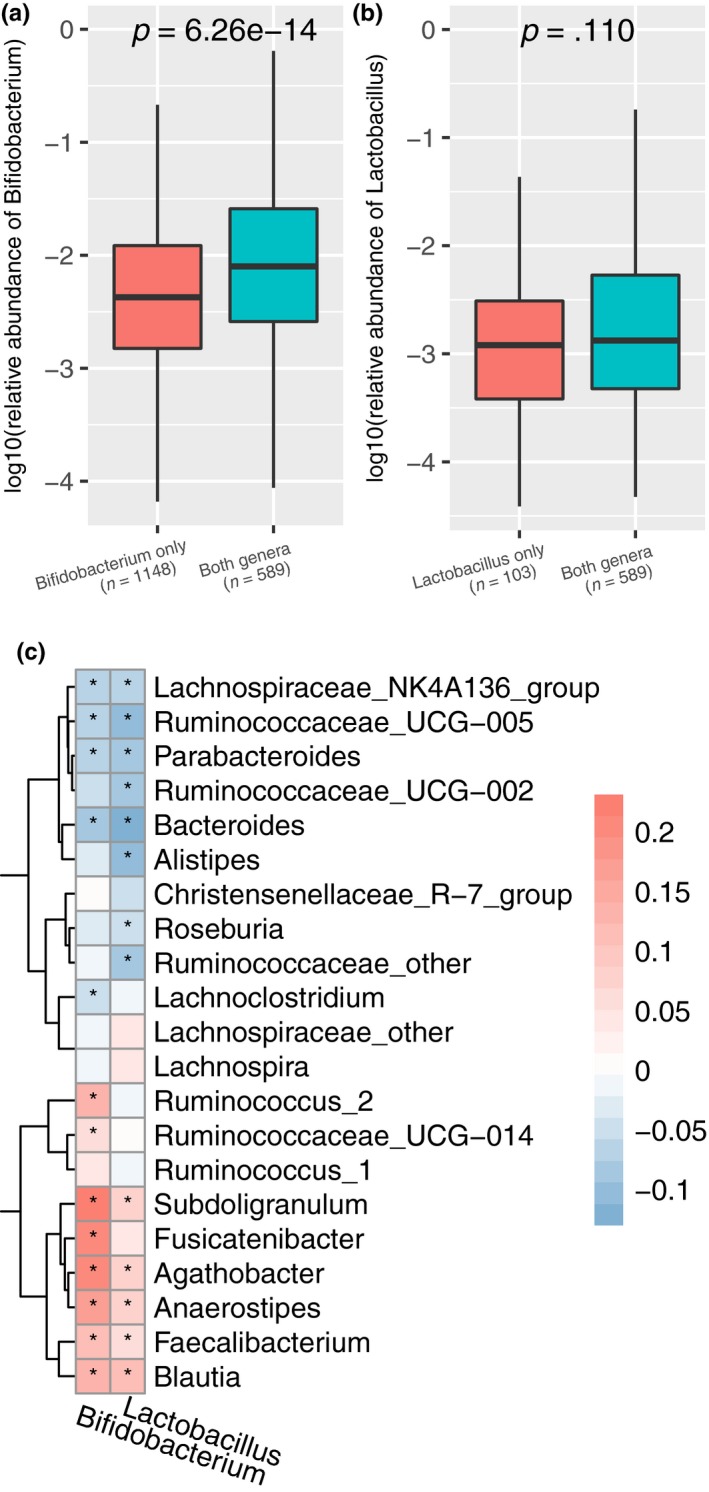
Cooccurrence of *Bifidobacterium* and *Lactobacillus* and correlation between these two genera and other dominant genera. (a) Relative abundance of *Bifidobacterium* in samples containing *Bifidobacterium* but not *Lactobacillus* or containing both genera. (b) Relative abundance of *Lactobacillus* in samples containing *Lactobacillus* but not *Bifidobacterium* or containing both genera. (c) Spearman's correlation between these two genera and other dominant genera. Red: positive correlation; blue: negative correlation; *, adjusted *p* < .05

**Figure A5:**
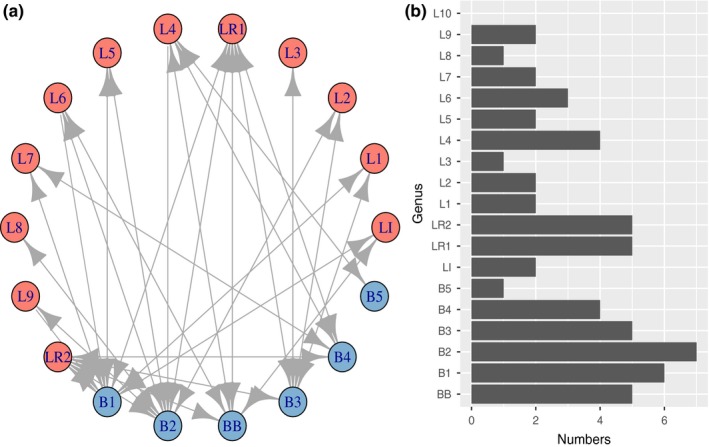
Cooccurrence of *Bifidobacterium* and *Lactobacillus* ASVs. (a) Network of cooccurrence of ASVs from different genera. The lines indicate a significantly increased relationship between one ASV belonging to *Bifidobacterium* and one ASV belonging to *Lactobacillus* (FDR < 0.1). L, *Lactobacillus*; B, *Bifidobacterium*; arrow: possible “promotion” between the two ASVs. (b) Number of promotions between one ASV belonging to one genus and another ASV belonging to the other genus

To explore the relationship between *Bifidobacterium* and *Lactobacillus*, we focused our analysis on the increase in these two genera when codetected. The relative abundance of *Bifidobacterium* was increased significantly when *Lactobacillus* was present (Figure 2a). At the same time, the relative abundance of *Lactobacillus* did not increase significantly when *Bifidobacterium* was present (Figure 2b). In addition, we found significantly increased levels of portions of *Bifidobacterium* and *Lactobacillus* ASVs when these genera were codetected (Figure A5). Considering the interinfluence between these two genera, we propose that these two genera also have a close connection with other dominant genera. We found that *Bifidobacterium* and *Lactobacillus* showed a positive correlation with *Blautia*, *Faecalibacterium*, *Anaerostipes*, *Agathobacter,* and *Subdoligranulum*, all of which are potential butyrate producers. Concomitantly, we also found a negative correlation of these two genera with some potential butyrate producers (Figure 2c) (Vital, Howe, & Tiedje, 2014). It can be argued that other factors exerting an effect on butyrate producers in the gut microbiota may exist.

**Figure 3:**
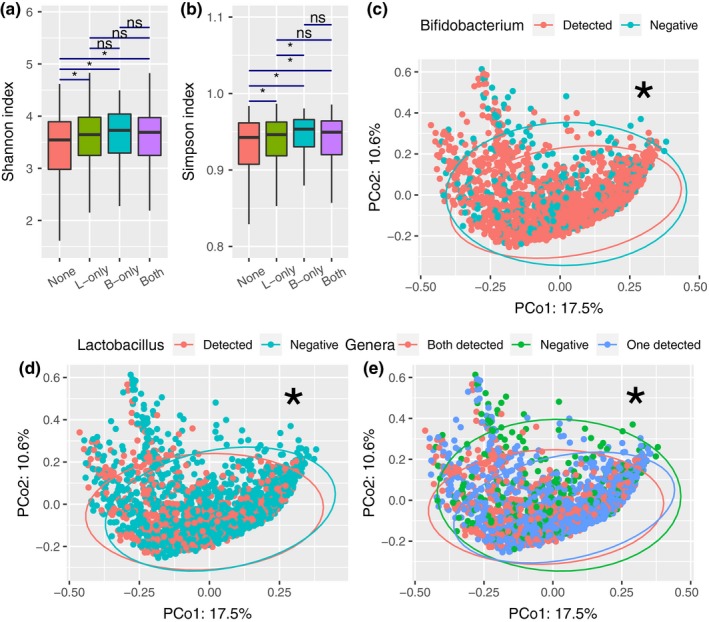
Alpha diversity and beta diversity of the 1,836 samples. Shannon index (a) and Simpson index (b) for the four groups. Statistical tests were performed using the Wilcoxon rank‐sum test. PCoA was based on Bray–Curtis dissimilarity considering the presence of *Bifidobacterium* (c), *Lactobacillus* (d), and the number of these two genera (e). *: *p* < .001 (PERMANOVA, permutation = 9,999)

**Figure A6:**
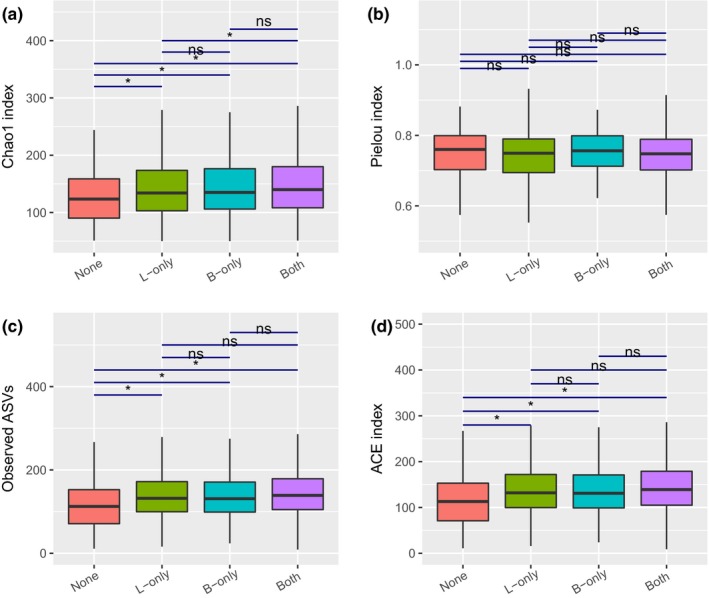
Different indexes of alpha diversity. (a) Chao1 index; (b) Pielou index; (c) Observed ASVs; and (d) ACE index

**Figure A7:**
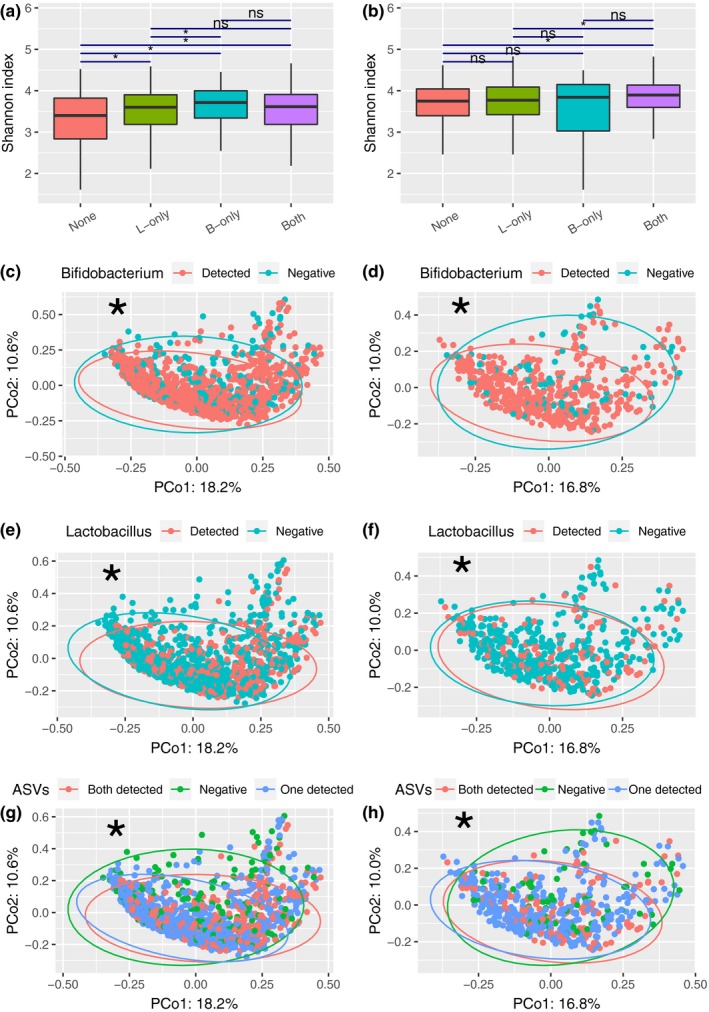
Alpha diversity and beta diversity of the sample from the USA and the UK. Shannon index for the USA (a) and the UK (b) among the four groups. Statistical tests were performed using the Wilcoxon rank‐sum test. PCoA was based on Bray–Curtis dissimilarity considering the presence of *Bifidobacterium* (c), *Lactobacillus* (e) for the USA sample. PCoA was based on Bray–Curtis dissimilarity considering the presence of *Bifidobacterium* (d) and *Lactobacillus* (f) for the UK sample. PCoA was based on Bray–Curtis dissimilarity considering the number of these two genera for the USA sample (g) and the UK sample (h). *: *p* < .001 (PERMANOVA, permutation = 9,999)

Furthermore, we compared the alpha diversity of the gut microbiota in the AGP dataset, with alpha diversity increasing as the number of codetected *Bifidobacterium* and *Lactobacillus* increased (Figure 3a,b and Figure A6). In addition, samples containing *Bifidobacterium* and not *Lactobacillus* showed a higher Simpson index than did those containing only *Lactobacillus*. The association between the two genera and the diversity of the gut microbiota was obvious for the US samples, but that for the UK samples was weaker (Figure A7). We visualized beta diversity by PCoA according to Bray–Curtis dissimilarities (Figure 3c‐e). An additional PERMANOVA analysis based on categorical variables of their abundance showed that the presence of *Bifidobacterium* and *Lactobacillus* was a significant factor in the variation of the gut microbiota (*p* < .001). Approximately 1% of the variance in beta diversity was explained by the presence of the two genera (*R* ^2^ = .010, .010, and .013, respectively), which is competitive with many microbiome covariates (Falony et al., 2016).

### 3.3 Robustness of microbial networks related to *Bifidobacterium* and *Lactobacillus*

**Figure 4:**
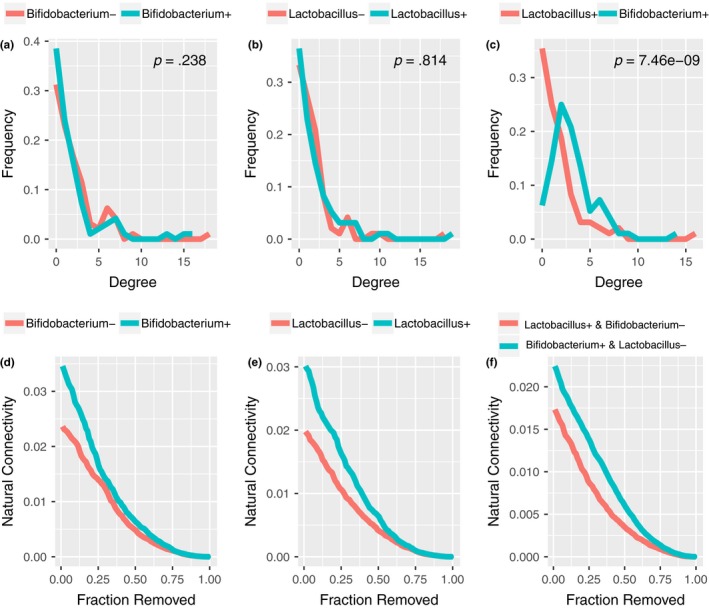
Microbial structure in relation to colonization. (a) Degree distribution of samples containing or not *Bifidobacterium*. (b) Degree distribution of samples containing or not *Lactobacillus*. (c) Degree distribution of samples containing *Bifidobacterium* but not *Lactobacillus* and samples containing *Lactobacillus* but not *Bifidobacterium*. (d) Natural connectivity is shown as a function of the size of the remaining network with the presence of *Bifidobacterium*. (e) Natural connectivity is shown as a function of the size of the remaining network with the presence of *Lactobacillus*. (f) Natural connectivity is shown as a function of the size of the remaining network of samples harboring *Bifidobacterium* present but no *Lactobacillus* and samples harboring *Lactobacillus* present but no *Bifidobacterium*. We performed node removals at random distribution of the natural connectivity

**Figure A8:**
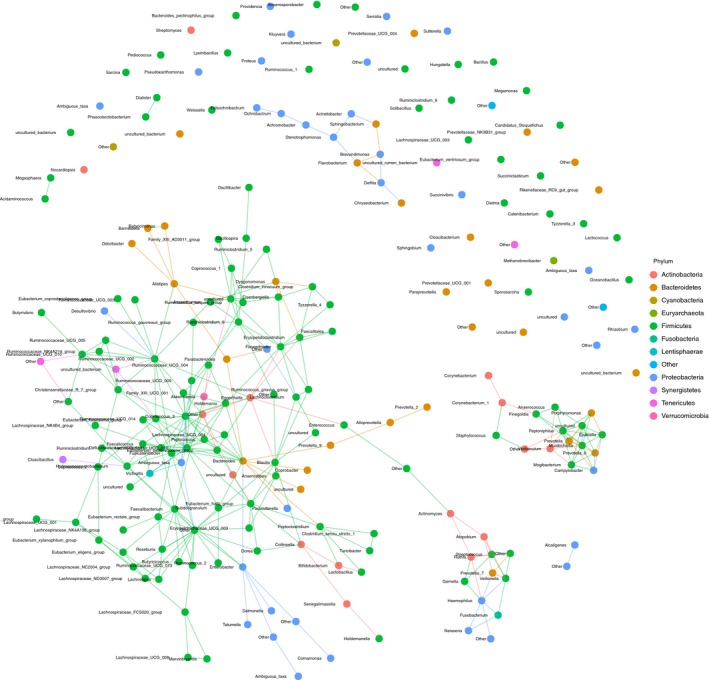
Cooccurrence network of microbial taxa detected in the AGP dataset. The different colors of the nodes represent different phyla

**Figure A9:**
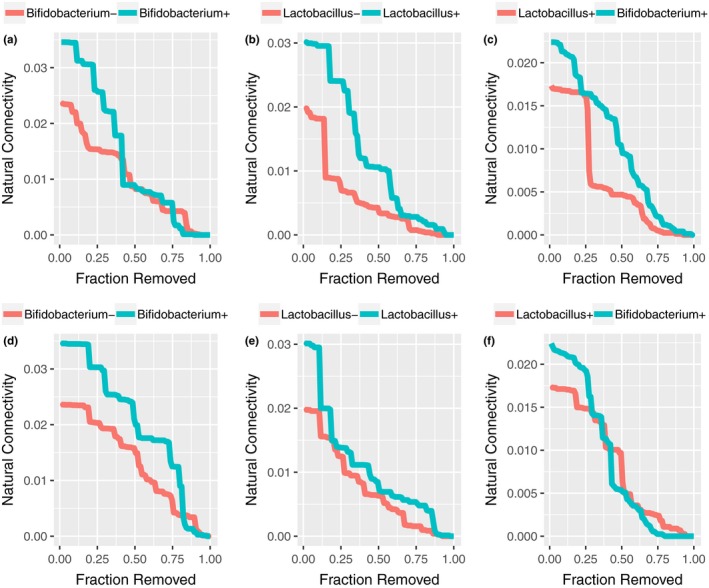
Microbial structure in relation to the presence of *Bifidobacterium* and *Lactobacillus*. Natural connectivity of the bacterial network with the presence of (a,d) *Bifidobacterium*, (b,e) *Lactobacillus,* and (c,f) only *Bifidobacterium* and only *Lactobacillus*. Node removals were ordered by the degree (a‐c) and betweenness (d‐f) of the natural connectivity

**Figure A10:**
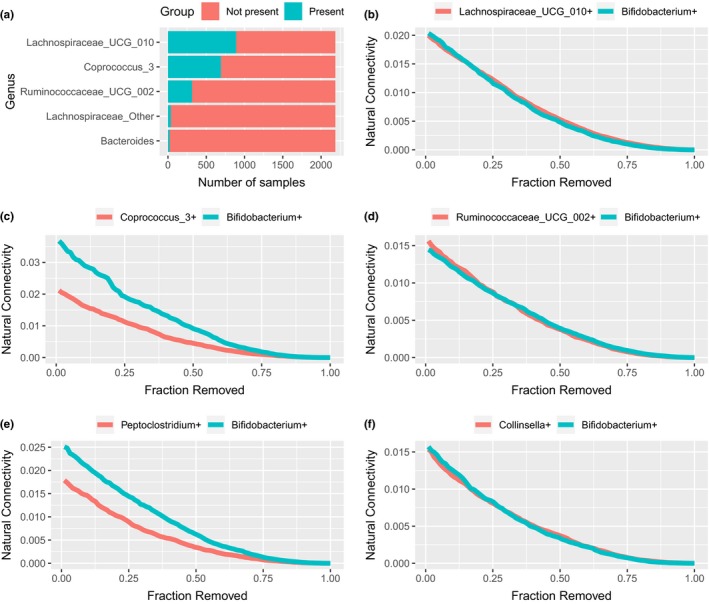
Microbial structure in relation to the colonization of *Bifidobacterium* and other genera. (a) Number of samples that have Top 5 highly interconnected genera. Natural connectivity of the network for comparisons between the presence of only *Bifidobacterium* and the presence of only *Lachnospiraceae_UCG_010* (b), *Coprococcus_*3 (c), *Ruminococcaceae_UCG_002* (d), *Peptoclostridium* (e), and *Collinsella* (f)

Analysis of the entire network constructed using the genus data from the AGP dataset showed that *Bifidobacterium* and *Lactobacillus* were not highly connected in the microbial network, suggesting that they were not keystone taxa for the cohort. However, notably, these two genera were connected to the largest cluster via *Peptoclostridium* and *Collinsella*; furthermore, *Bifidobacterium* and *Lactobacillus* were connected to each other (Figure A8). To further explore the effect of *Bifidobacterium* and *Lactobacillus* on the robustness of the microbial network, we performed three comparisons of the microbial community structure, considering the presence of these two genera (Figure 4). The degree statistics for the networks containing or not containing *Bifidobacterium* and *Lactobacillus* were not statistically significant (*p* = .238 and *p* = .814, respectively). However, the bacterial network of samples containing *Bifidobacterium* but not *Lactobacillus* showed higher statistics than did those only containing *Lactobacillus* (Figure 4c, *p* = 7.46 × 10^−9^). We then compared the resilience of the networks to degree disturbance using random node removal to simulate an “attack” on the networks (Mahana et al., 2016). With the absence of either *Bifidobacterium* or *Lactobacillus*, the natural connectivity of the microbial network decreased faster compared to the connectivity that when either of these genera were present (Figure 4d,e). In addition, the microbial network constructed for the samples containing *Lactobacillus* but not *Bifidobacterium* decreased faster compared with the connectivity when *Bifidobacterium* but not *Lactobacillus* was present (Figure 4f). Node removals ordered by the degree and betweenness of the natural connectivity suggested the same results (Figure A9). Taken together, these results indicate that the presence of *Bifidobacterium* and *Lactobacillus*, especially *Bifidobacterium*, was more important for maintaining the robustness of the bacterial network. To further test the importance of *Bifidobacterium* to the robustness of the gut microbiota, we compared the genus with other genera based on the number of connections shown in the cooccurrence network (Table A8 in Appendix 1). Among the top 5 highly interconnected genera, there are not enough samples to build a bacterial network for *Bacteroides* and *Lachnospiraceae_Other* (Figure A10a)*.* The results showed that the ability of *Bifidobacterium* to sustain the gut microbiota robustness under attack was comparable to the most frequently connected genus examined (Figure A10b‐d).

### 3.4 The effect of *Bifidobacterium* and *Lactobacillus* on the gut microbiota

**Figure 5:**
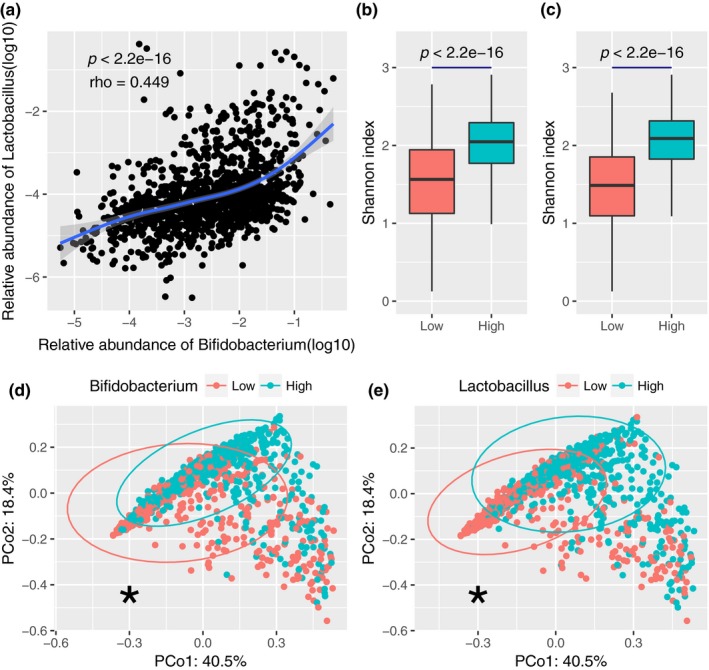
Validation of the results obtained with the AGP dataset using the NBT dataset. (a) Correlation between the relative abundances of *Bifidobacterium* and *Lactobacillus*. (b) Shannon index in the samples containing either only *Bifidobacterium* or both genera. (c) Relative abundance of *Lactobacillus* in the samples containing either only *Lactobacillus* or both genera. (d) PCoA based on Bray–Curtis dissimilarity considering the presence of *Bifidobacterium*. (e) PCoA based on Bray–Curtis dissimilarity considering the presence of *Lactobacillus*. *: p‐value < 0.001 (PERMANOVA)

**Figure A11:**
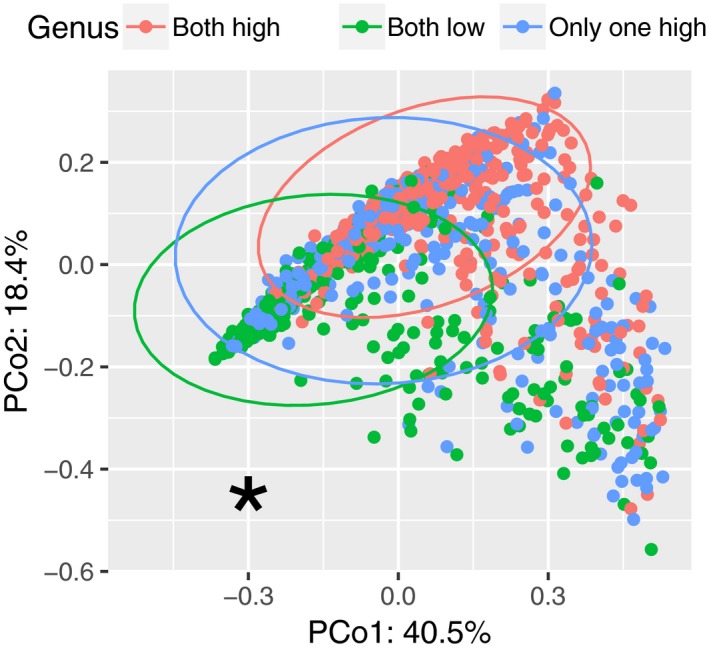
PCoA based on the Bray–Curtis dissimilarity distance considering the abundance of *Bifidobacterium* and *Lactobacillus*. *: p‐value < 0.001 (PERMANOVA)

**Figure A12:**
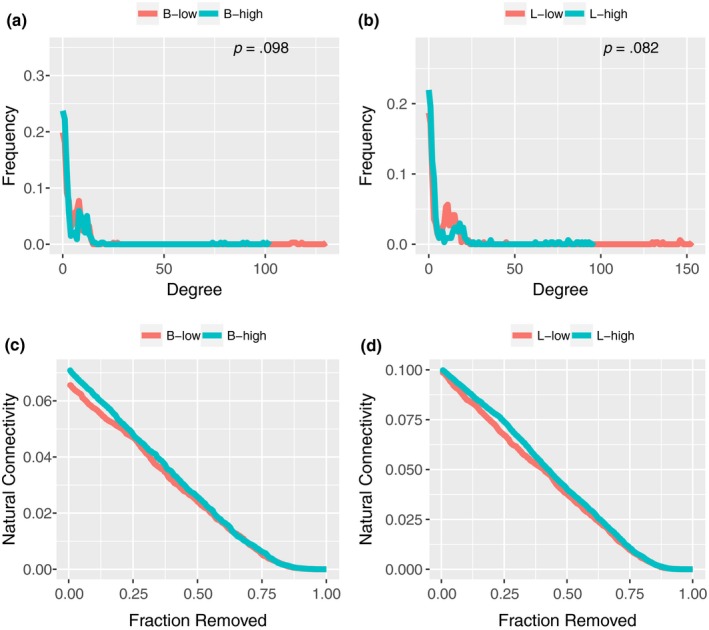
Degree distribution and natural connectivity. (a) Degree distribution of samples with a higher abundance of *Bifidobacterium* and samples with a lower abundance of *Bifidobacterium*. (b) Degree distribution of samples with a higher abundance of *Lactobacillus* and samples with a lower abundance of *Lactobacillus*. (c) Node removals were ordered at a random distribution of the natural connectivity for the presence or absence of *Bifidobacterium*. (d) Node removals were ordered at a random distribution of the natural connectivity for the presence or absence of *Lactobacillus*. B‐low, lower relative abundance of *Bifidobacterium*; B‐high, higher relative abundance of *Bifidobacterium*; L‐Low, lower relative abundance of *Lactobacillus*; L‐high, higher relative abundance of *Lactobacillus*

We validated the influence of *Bifidobacterium* and *Lactobacillus* on the gut microbiota using genus data from the NBT dataset, which were annotated based on reference genomes with a similarity of >85% at the genus level (Li et al., 2014). Due to the sequencing depth, all 1,267 samples showed positive results for the two genera (Table A9 in Appendix 1). Therefore, we divided the samples into two groups, a higher group and a lower group, according to the median value of relative abundance. Spearman's correlation analysis showed a positive correlation between the relative abundance of the two genera (rho = .449, *p* < 2.2 × 10^−16^, Figure 5a). In addition, the samples with higher relative abundances of *Bifidobacterium* and *Lactobacillus* showed higher alpha diversities, similar to the result found on the AGP dataset (Figure 5b,c). There was also a significant association between beta diversity and a higher relative abundance of *Bifidobacterium* or *Lactobacillus* (Figure 5d,e and Figure A11). Natural connectivity decreased faster in the group with a lower relative abundance of *Bifidobacterium* or *Lactobacillus* than in the group with a higher relative abundance, though this was not as noticeable as seen in the results for the AGP dataset (Figure A12).

### 3.5 The abundance of *Bifidobacterium* is associated with demographic features, lifestyle, and diseases

**Figure 6:**
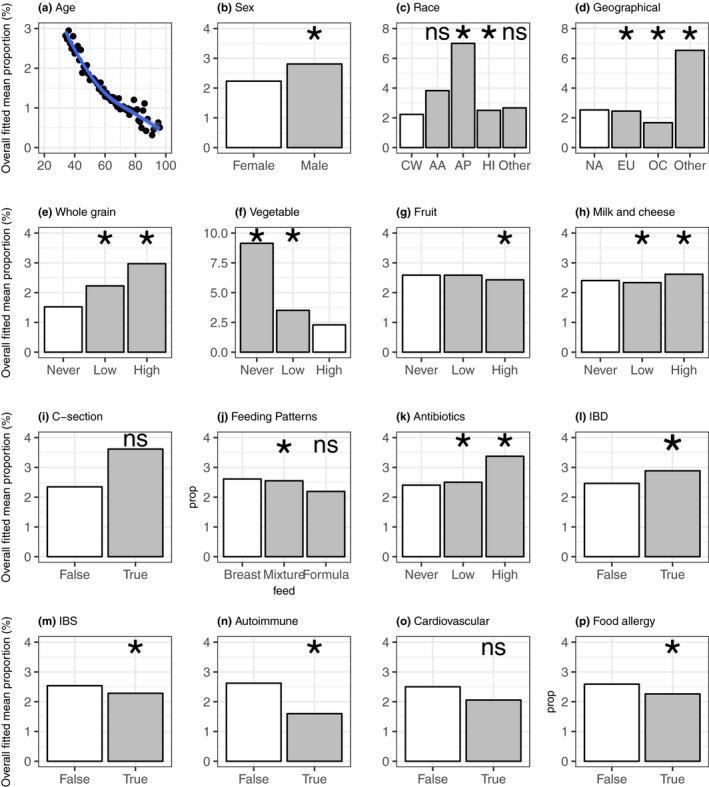
Predicted relationships between *Bifidobacterium* abundance and host features based on the ZINB model. The overall fitted mean proportions (%) of *Bifidobacterium* and age (a); sex (b); race (c); geographical location (d); whole‐grain consumption (e); vegetable consumption (f); fruit consumption (g); milk and cheese consumption (h); C‐section (i); fermented plant consumption (i); feeding patterns (j); antibiotic exposure (k); IBD (l); IBS (m); autoimmune disease (n); cardiovascular disease (o); and food allergy (p). White bar: reference; gray bar: comparisons; race (CW, Caucasian White; AA, African‐American; AP, Asian‐Pacific; and HI, Hispanic); geographical (NA, North America; EU, Europe; and OC, Oceania); *: significance in at least in one part of the ZINB model (*p* < .05); ns: not significant in two parts of the ZINB model (*p* > .05)

As shown above, *Bifidobacterium* displayed a closer connection with the diversity and robustness of the gut microbiota than *Lactobacillus*, and we then focused on exploring the impacting factors related to the abundance of *Bifidobacterium*. To better understand the association between *Bifidobacterium* and background information, we included 16 factors with sufficient records to identify potential associations with the abundance of *Bifidobacterium* using all samples from the AGP dataset. The fitted ZINB model was constructed based on all 16 variables in one model on which they determined significance. We found many factors to be significantly associated with the relative abundance of *Bifidobacterium* (Table A10 in Appendix 1). For example, the relative abundance of *Bifidobacterium* was associated with demographic features included in the present study, namely, age, sex, race, and geographical location (Figure 6a‐d). In terms of lifestyle, we found that whole‐grain consumption, milk, and cheese were associated with an increased abundance of *Bifidobacterium*, though a high frequency of vegetables and fruits consumption negatively affected the abundance of *Bifidobacterium* (Figure 6e‐h). Breasting feeding in infants showed a close connection with a higher abundance of *Bifidobacterium*, even though our cohort consisted of adults (Figure 6j). Notably, a high relative abundance of *Bifidobacterium* was associated with IBD and recent antibiotic exposure (Figure 6k,l). However, people with IBS, autoimmune disease, and food allergy had a lower relative abundance of *Bifidobacterium* than did unaffected individuals (Figure 6m,n,p). These results also showed that the relative abundance of *Bifidobacterium* was not associated with cardiovascular disease or C‐section (Figure 6i,o).

## 4 DISCUSSION

We found the following through analysis of the AGP dataset: (1) *Bifidobacterium* was a common genus, but *Lactobacillus* was not; (2) the abundances of *Bifidobacterium* and *Lactobacillus* were positively correlated, especially at the ASV level; (3) samples containing the two genera showed higher alpha diversity; (4) *Bifidobacterium* was more helpful than *Lactobacillus* in sustaining the robustness of the gut microbiota based on the inferred microbial network; (5) demographic features, lifestyle, and diseases were closely connected with the relative abundance of *Bifidobacterium*.

Dominant taxa with large biomasses or major energy transformations might influence a broad array of processes, such as denitrification or organic matter decomposition (Banerjee et al., 2018). Based on the results of our analysis, *Bifidobacterium* had a higher relative abundance and a wider prevalence than *Lactobacillus*, indicating a stronger influence on gut microbiota processes. The *Bifidobacterium*‐mediated effect is an important issue that needs to be addressed in relation to strain‐specific beneficial properties (Presti et al., 2015). Although we explored each ASV to improve classification accuracy, the lengths of the sequenced amplicons made it difficult to classify them at the species level. Furthermore, our results suggested that the most abundant ASV (*Bifidobacterium_*1) belonging to *Bifidobacterium* showed a higher identity to *B. longum*, *B. adolescentis,* and *B. breve*, which are frequently used probiotics, despite an inability to analyze the data at the species level.

Our results suggested that the relative abundance of *Bifidobacterium* increased when *Lactobacillus* was present. The cooccurrence network and the NBT dataset also showed a close correlation between these two genera. These observations suggest that cooperation may exist between these two genera. This relationship may explain why multistrain probiotics appear to show greater efficacy than single‐strain probiotics (Chapman, Gibson, & Rowland, 2011). In addition, many factors could lead to the same observation, such as taking probiotics and dairy products containing *Bifidobacterium* and *Lactobacillus*. Cross‐feeding interactions were studied between selected strains of *Bifidobacterium*/*Lactobacillus* and butyrate‐producing bacteria that consume lactate (Moens, Verce, & De Vuyst, 2017). Our results verified that the positive correlation between *Bifidobacterium*/*Lactobacillus* and butyrate‐producing bacteria may be one of the beneficial roles played by these two genera in the host.

The present study confirmed that the presence of these two genera is associated with higher alpha diversity. Interestingly, *Bifidobacterium* has a strong effect on the alpha diversity of the gut microbiota through mechanisms that may include starch‐degrading activity (Ryan, Fitzgerald, & van Sinderen, 2006). Moreover, our results suggested that *Bifidobacterium* and *Lactobacillus* are not only associated with alpha diversity but may also be related to the microbial structure. A previous study indicated that the fish gut microbiota was less affected by spatial differences resulting from environmental factors via increases in the abundance of a certain strain (Giatsis et al., 2016). This finding indicates that some types of bacteria may help sustain the robustness of the gut microbiota. Indeed, according to the results of our present study, *Bifidobacterium* helps sustain global network connectivity. *Bifidobacterium* helps in the resistance of the microbiota to the effects of other factors, such as a high‐fat diet and antibiotics (Kristensen et al., 2016). Moreover, comparison with another six genera proved the important role of *Bifidobacterium* in the gut microbiota. Microbial keystone taxa are highly connected taxa that, individually or together, exert considerable influence on microbiome structure and function (Banerjee et al., 2018). Nonetheless, *Bifidobacterium* did not exhibit high connectivity with other genera, indicating that they may not be keystone taxa. However, according to Angulo's study, manipulation of driver species, which are not always highly interconnected, may control the entire network (Angulo, Moog, & Liu, 2019). Therefore, *Bifidobacterium* and *Lactobacillus* might be potential drivers of the bacterial network. In addition, the role of *Peptoclostridium* and *Collinsella* in the gut microbiota still needs to be explored, as these genera were the only two found to be closely connected with *Bifidobacterium* and *Lactobacillus*.

Considering the increasing global incidence of many diseases, changes in lifestyle and diet have been proposed to contribute to disease emergence by altering gut microbial ecology (Blaser, 2006), and many strains of *Bifidobacterium* have been used to improve health. However, it is uncertain whether intake of *Bifidobacterium* strains can ameliorate the symptoms of conditions such as IBS (Cozmapetruţ et al., 2017), allergy (Mennini et al., 2017), and diarrhea (Laursen et al., 2017), even in clinical trials. These findings suggest that the association between disease and *Bifidobacterium* is questionable. In the present study, we found that the relative abundance of *Bifidobacterium* is under the influence of demographic features. Indeed, it has been reported that age, geography, and ethnic origins are factors that influence the abundance of *Bifidobacterium* (Deschasaux et al., 2018; Kato et al., 2017). In terms of lifestyle, we observed that higher consumption of whole grains and dairy products was associated with a higher abundance of *Bifidobacterium* in the gut microbiota (Martinez et al., 2013). However, C‐section did not appear to influence the abundance of *Bifidobacterium* in adults, even though it is associated with *Bifidobacterium* colonization in infants (Hesla et al., 2014). This finding suggests that the lifelong effect of C‐section on *Bifidobacterium* is unlikely. The decreased abundance of *Bifidobacterium* related to higher consumption of vegetables and fruits may be due to other factors not included in the present study, which is a limitation of the present study. A small sample number may be another factor leading to this unexpected result (Table A1 in Appendix 1). Surprisingly, exposure to antibiotics increased the relative abundance of *Bifidobacterium*, a finding that needs to be investigated further. One plausible explanation for this increase could be the use of probiotics considering *Bifidobacterium*_1 showed identity to the species commonly used as probiotics (Figure A3); however, this information was not included in the metadata. Increased relative abundance of *Bifidobacterium* in the gut microbiota may be helpful for controlling IBS (Han, Wang, Seo, & Kim, 2017), autoimmune disease (Uusitalo et al., 2016), and food allergy (Mennini et al., 2017), as the relative abundance of *Bifidobacterium* was lower in patients with these conditions than in unaffected individuals. However, all these results together with those we presented here are mostly correlation analyses; the relationship between *Bifidobacterium* and human diseases and if *Bifidobacterium* bacteria could be a treatment option still needs to be revealed.

We note the following limitations of the present study: This study was only performed on two datasets, not on diverse geographic origins; the contribution of *Bifidobacterium* to the diversity and robustness was only analyzed by comparison with *Lactobacillus* and not other genera; the background information was not sufficiently detailed to allow a solid conclusion to be drawn, with some ambiguous information; many factors influence the relative abundance of *Bifidobacterium*, which makes it difficult to interpret the results of the association between lifestyle and the relative abundance of *Bifidobacterium*; there may be more important bacteria other than *Bifidobacterium* and *Lactobacillus*, which was not evaluated in the present study.

## 5 CONCLUSIONS

In summary, our results showed a close connection between *Bifidobacterium* and *Lactobacillus*. The genus *Bifidobacterium* was important for the diversity and robustness of the gut microbiota. Increasing the intake of whole grains and dairy products may be a good way to increase the abundance of *Bifidobacterium*.

## CONFLICT OF INTEREST

None declared.

## AUTHOR CONTRIBUTIONS

Yuqing Feng contributed to conceptualization; Yuqing Feng, Na Lyu, Fei Liu, and Shihao Liang contributed to formal analysis; Baoli Zhu contributed to funding acquisition; Yuqing Feng, Yunfeng Duan contributed to writing‐original draft preperation; Zhenjiang Xu contributed to writing‐review and editing.

## ETHICAL APPROVAL

None required.

## Data Availability

All data used for this paper is available at ebi.ac.uk/ena (accession # https://www.ebi.ac.uk/ena/data/view/PRJEB11419) for the AGP dataset and meta.genomics.cn/meta/dataTools for the NBT dataset. The R scripts used for analysis in this paper are available in the following link: https://doi.org/10.6084/m9.figshare.9756599.v1.

## APPENDIX 1

**Table A1:** Descriptive statistics for candidate for 11,744 fecal samples

Covariates	All samples	
Continuous variable	Mean ± *SD*	No. of missing
Age	45.18 ± 17.66	483
Categorical covariate	No. of records	No. of missing
Sex		
Female	5,947	557
Male	5,240	
Race		
Caucasian	10,201	321
African American	86	
Asian/Pacific Islander	565	
Hispanic	239	
Others	332	
Geographic location		
North America	8,542	28
Europe	2,824	
Oceania	302	
Others	48	
Alcohol consumption		
False	1,954	3,402
True	6,388	
Fermented plant frequency		
Never	2,710	3,915
Low frequency	3,685	
High frequency	1,434	
Milk and cheese frequency		
Never	1,206	3,763
Low frequency	2,826	
High frequency	3,949	
Whole grain frequency		
Never	1,020	3,841
Low frequency	3,249	
High frequency	3,634	
Fruit frequency		
Never	438	3,783
Low frequency	2,634	
High frequency	4,889	
Feeding patterns		
Primarily breast milk	3,744	4,826
Primarily infant formula	1,881	
A mixture of breast milk and formula	1,293	
Born by C‐section		
FALSE	9,759	819
TRUE	1,166	
Vegetable consumption frequency		
Never	65	3,771
Low frequency	964	
High frequency	6,944	
Last exposure to antibiotics		
Over 1 year	7,672	409
Low frequency	3,023	
High frequency	640	
Food allergy		
False	5,493	1
True	6,250	
IBD		
False	10,408	857
True	479	
SIBO		
False	7,203	3,996
True	545	
IBS		
False	6,256	3,879
True	1,609	
Autoimmune disease		
False	6,864	3,849
True	1,031	
Cardiovascular disease		
False	7,668	3,795
True	281	
Mental illness		
False	3,681	7,477
True	586	

**Table A2:** Inclusion criteria for individuals whose samples were used in the analyses

Category	Criteria
Age (year)	Exclude infants (0 ≤ age ≤ 1)
BMI	18.5–30.0
Last exposure to antibiotics	Over 1 month
Acid reflux	No
Appendix removed	No
Autoimmune disease	No
Cancer	No
Cardiovascular disease	No
Clinical condition	No
IBD	No
IBS	No
Liver disease	No
Lung disease	No
Mental Illness	No
PKU	No
Pregnant	No
SIBO	No

**Table A3:** Workflow of American Gut Project data processing

Steps	Contents
Step 1	Downloaded 19,327 samples (25 Jan. 2018)
Step 2	Excluded non‐fecal samples: 15,259 fecal samples left
Step 3	12,127 fecal samples with over 10,000 reads
Step 4	11,744 samples passed the quality control of DADA2
Step 5	Delete the ASV with a distribution of less 1% and not belong to bacteria
Step 6	Delete blooming bacteria
Step 7	Excluded samples with diseases

**Table A4:** Relative abundance of dominant genera in 2,186 samples

Genus	Relative abundance of dominant genera
Bacteria|Bacteroidetes|Bacteroidia|Bacteroidales|Bacteroidaceae|Bacteroides	22.7%
Bacteria|Firmicutes|Clostridia|Clostridiales|Ruminococcaceae|Faecalibacterium	7.9%
Bacteria|Firmicutes|Clostridia|Clostridiales|Lachnospiraceae|Other	3.7%
Bacteria|Firmicutes|Clostridia|Clostridiales|Lachnospiraceae|Agathobacter	3.3%
Bacteria|Firmicutes|Clostridia|Clostridiales|Lachnospiraceae|Blautia	2.9%
Bacteria|Bacteroidetes|Bacteroidia|Bacteroidales|Rikenellaceae|Alistipes	2.8%
Bacteria|Firmicutes|Clostridia|Clostridiales|Ruminococcaceae|Subdoligranulum	2.3%
Bacteria|Bacteroidetes|Bacteroidia|Bacteroidales|Tannerellaceae|Parabacteroides	2.2%
Bacteria|Firmicutes|Clostridia|Clostridiales|Ruminococcaceae|Ruminococcaceae_UCG‐014	2.1%
Bacteria|Firmicutes|Clostridia|Clostridiales|Ruminococcaceae|Ruminococcaceae_UCG‐002	2.1%
Bacteria|Firmicutes|Clostridia|Clostridiales|Christensenellaceae|Christensenellaceae_R‐7_group	1.6%
Bacteria|Actinobacteria|Actinobacteria|Bifidobacteriales|Bifidobacteriaceae|Bifidobacterium	1.4%
Bacteria|Firmicutes|Clostridia|Clostridiales|Lachnospiraceae|Roseburia	1.3%
Bacteria|Firmicutes|Clostridia|Clostridiales|Ruminococcaceae|Other	1.2%
Bacteria|Firmicutes|Clostridia|Clostridiales|Ruminococcaceae|Ruminococcus_2	1.2%
Bacteria|Firmicutes|Clostridia|Clostridiales|Lachnospiraceae|Lachnospiraceae_NK4A136_group	1.1%
Bacteria|Firmicutes|Clostridia|Clostridiales|Ruminococcaceae|Ruminococcus_1	0.9%
Bacteria|Firmicutes|Clostridia|Clostridiales|Lachnospiraceae|Anaerostipes	0.8%
Bacteria|Firmicutes|Clostridia|Clostridiales|Ruminococcaceae|Ruminococcaceae_UCG‐005	0.8%
Bacteria|Firmicutes|Clostridia|Clostridiales|Lachnospiraceae|Lachnospira	0.8%
Bacteria|Firmicutes|Clostridia|Clostridiales|Lachnospiraceae|Fusicatenibacter	0.7%
Bacteria|Firmicutes|Clostridia|Clostridiales|Lachnospiraceae|Lachnoclostridium	0.6%

**Table A5:** Relative abundance of dominant genera in samples from USA

Genus	Relative abundance of dominant genera
Bacteria|Bacteroidetes|Bacteroidia|Bacteroidales|Bacteroidaceae|Bacteroides	24.0%
Bacteria|Firmicutes|Clostridia|Clostridiales|Ruminococcaceae|Faecalibacterium	7.7%
Bacteria|Firmicutes|Clostridia|Clostridiales|Lachnospiraceae|Other	3.9%
Bacteria|Firmicutes|Clostridia|Clostridiales|Lachnospiraceae|Agathobacter	3.4%
Bacteria|Firmicutes|Clostridia|Clostridiales|Lachnospiraceae|Blautia	3.1%
Bacteria|Bacteroidetes|Bacteroidia|Bacteroidales|Rikenellaceae|Alistipes	2.7%
Bacteria|Bacteroidetes|Bacteroidia|Bacteroidales|Tannerellaceae|Parabacteroides	2.3%
Bacteria|Firmicutes|Clostridia|Clostridiales|Ruminococcaceae|Subdoligranulum	2.2%
Bacteria|Firmicutes|Clostridia|Clostridiales|Ruminococcaceae|Ruminococcaceae_UCG‐002	1.8%
Bacteria|Firmicutes|Clostridia|Clostridiales|Lachnospiraceae|Roseburia	1.4%
Bacteria|Actinobacteria|Actinobacteria|Bifidobacteriales|Bifidobacteriaceae|Bifidobacterium	1.3%
Bacteria|Firmicutes|Clostridia|Clostridiales|Christensenellaceae|Christensenellaceae_R‐7_group	1.3%
Bacteria|Firmicutes|Clostridia|Clostridiales|Ruminococcaceae|Other	1.1%
Bacteria|Firmicutes|Clostridia|Clostridiales|Lachnospiraceae|Lachnospiraceae_NK4A136_group	1.1%
Bacteria|Firmicutes|Clostridia|Clostridiales|Lachnospiraceae|Anaerostipes	0.9%
Bacteria|Firmicutes|Clostridia|Clostridiales|Lachnospiraceae|Lachnospira	0.9%
Bacteria|Firmicutes|Clostridia|Clostridiales|Ruminococcaceae|Ruminococcus_1	0.8%
Bacteria|Firmicutes|Clostridia|Clostridiales|Lachnospiraceae|Fusicatenibacter	0.8%
Bacteria|Firmicutes|Clostridia|Clostridiales|Lachnospiraceae|Lachnoclostridium	0.7%
Bacteria|Firmicutes|Clostridia|Clostridiales|Ruminococcaceae|Ruminococcaceae_UCG‐005	0.7%
Bacteria|Firmicutes|Erysipelotrichia|Erysipelotrichales|Erysipelotrichaceae|Erysipelotrichaceae_UCG‐003	0.6%

**Table A6:** Relative abundance of dominant genera in samples from UK

Genus	Relative abundance of dominant genera
Bacteria|Bacteroidetes|Bacteroidia|Bacteroidales|Bacteroidaceae|Bacteroides	20.0%
Bacteria|Firmicutes|Clostridia|Clostridiales|Ruminococcaceae|Faecalibacterium	8.4%
Bacteria|Firmicutes|Clostridia|Clostridiales|Lachnospiraceae|Other	3.3%
Bacteria|Bacteroidetes|Bacteroidia|Bacteroidales|Rikenellaceae|Alistipes	3.1%
Bacteria|Firmicutes|Clostridia|Clostridiales|Lachnospiraceae|Agathobacter	3.1%
Bacteria|Firmicutes|Clostridia|Clostridiales|Ruminococcaceae|Ruminococcaceae_UCG‐014	3.1%
Bacteria|Firmicutes|Clostridia|Clostridiales|Ruminococcaceae|Ruminococcaceae_UCG‐002	2.9%
Bacteria|Firmicutes|Clostridia|Clostridiales|Lachnospiraceae|Blautia	2.4%
Bacteria|Firmicutes|Clostridia|Clostridiales|Ruminococcaceae|Subdoligranulum	2.2%
Bacteria|Firmicutes|Clostridia|Clostridiales|Christensenellaceae|Christensenellaceae_R‐7_group	2.1%
Bacteria|Verrucomicrobia|Verrucomicrobiae|Verrucomicrobiales|Akkermansiaceae|Akkermansia	2.1%
Bacteria|Bacteroidetes|Bacteroidia|Bacteroidales|Tannerellaceae|Parabacteroides	2.0%
Bacteria|Firmicutes|Clostridia|Clostridiales|Ruminococcaceae|Other	1.6%
Bacteria|Tenericutes|Mollicutes|Mollicutes_RF39|Other|Other	1.4%
Bacteria|Firmicutes|Clostridia|Clostridiales|Ruminococcaceae|Ruminococcus_2	1.4%
Bacteria|Actinobacteria|Actinobacteria|Bifidobacteriales|Bifidobacteriaceae|Bifidobacterium	1.3%
Bacteria|Firmicutes|Clostridia|Clostridiales|Ruminococcaceae|Ruminococcaceae_UCG‐005	1.2%
Bacteria|Firmicutes|Clostridia|Clostridiales|Lachnospiraceae|Lachnospiraceae_NK4A136_group	1.1%
Bacteria|Firmicutes|Clostridia|Clostridiales|Lachnospiraceae|Roseburia	1.1%
Bacteria|Firmicutes|Clostridia|Clostridiales|Ruminococcaceae|Ruminococcus_1	1.0%
Bacteria|Firmicutes|Clostridia|Clostridiales|Lachnospiraceae|Coprococcus_2	0.9%
Bacteria|Firmicutes|Clostridia|Clostridiales|Ruminococcaceae|Ruminococcaceae_NK4A214_group	0.7%

**Table A7:** Distribution of *Bifidobacterium* and *Lactobacillus*

ASV_ID	No. of the ASV present	Ratio of the ASV present
*Bifidobacterium bifidum*	198	9.1%
*Bifidobacterium_*1	1,525	69.8%
*Bifidobacterium_*2	445	20.4%
*Bifidobacterium_*3	190	8.7%
*Bifidobacterium_*4	42	1.9%
*Bifidobacterium_*5	30	1.4%
*Lactobacillus iners*	71	3.2%
*Lactobacillus ruminis_*1	102	4.7%
*Lactobacillus ruminis_*2	40	1.8%
*Lactobacillus_*1	206	9.4%
*Lactobacillus_*2	140	6.4%
*Lactobacillus_*3	99	4.5%
*Lactobacillus_*4	55	2.5%
*Lactobacillus_*5	75	3.4%
*Lactobacillus_*6	33	1.5%
*Lactobacillus_*7	26	1.2%
*Lactobacillus_*8	38	1.7%
*Lactobacillus_*9	38	1.7%
*Lactobacillus_*10	21	1.0%
*Bifidobacterium*	1,737	79.5%
*Lactobacillus*	692	31.7%

**Table A8:** Frequency of the genera connected analyzed by the bacterial network

No of the node connected	Phylum	Class	Order	Family	Genus
20	Firmicutes	Clostridia	Clostridiales	Lachnospiraceae	Lachnospiraceae_UCG_010
19	Firmicutes	Clostridia	Clostridiales	Lachnospiraceae	Other
19	Firmicutes	Clostridia	Clostridiales	Lachnospiraceae	Coprococcus_3
15	Firmicutes	Clostridia	Clostridiales	Ruminococcaceae	Ruminococcaceae_UCG_002
15	Bacteroidetes	Bacteroidia	Bacteroidales	Bacteroidaceae	Bacteroides
12	Firmicutes	Clostridia	Clostridiales	Defluviitaleaceae	Defluviitaleaceae_UCG_011
11	Firmicutes	Clostridia	Clostridiales	Lachnospiraceae	Eubacterium_hallii_group
11	Firmicutes	Clostridia	Clostridiales	Family_XI	Peptoniphilus
10	Firmicutes	Clostridia	Clostridiales	Ruminococcaceae	Faecalibacterium
10	Firmicutes	Clostridia	Clostridiales	Ruminococcaceae	Flavonifractor
10	Firmicutes	Clostridia	Clostridiales	Ruminococcaceae	uncultured
9	Firmicutes	Clostridia	Clostridiales	Ruminococcaceae	Ruminiclostridium_9
9	Actinobacteria	Coriobacteriia	Coriobacteriales	Coriobacteriaceae	Other
8	Firmicutes	Clostridia	Clostridiales	Ruminococcaceae	Hydrogenoanaerobacterium
8	Actinobacteria	Coriobacteriia	Coriobacteriales	Coriobacteriaceae	Eggerthella
8	Firmicutes	Clostridia	Clostridiales	Ruminococcaceae	Ruminococcaceae_UCG_010
8	Firmicutes	Clostridia	Clostridiales	Ruminococcaceae	Ruminococcaceae_UCG_014
8	Firmicutes	Erysipelotrichia	Erysipelotrichales	Erysipelotrichaceae	Holdemania
8	Bacteroidetes	Bacteroidia	Bacteroidales	Prevotellaceae	Prevotella
8	Firmicutes	Clostridia	Clostridiales	Christensenellaceae	Christensenellaceae_R7_group
8	Firmicutes	Clostridia	Clostridiales	Family_XI	Murdochiella
8	Firmicutes	Clostridia	Clostridiales	Family_XIII	uncultured
8	Firmicutes	Clostridia	Clostridiales	Lachnospiraceae	Blautia
7	Proteobacteria	Gammaproteobacteria	Enterobacteriales	Enterobacteriaceae	Enterobacter
7	Proteobacteria	Gammaproteobacteria	Pasteurellales	Pasteurellaceae	Haemophilus
7	Firmicutes	Clostridia	Clostridiales	Lachnospiraceae	Fusicatenibacter
6	Firmicutes	Clostridia	Clostridiales	Lachnospiraceae	Eubacterium_oxidoreducens_group
6	Firmicutes	Clostridia	Clostridiales	Peptostreptococcaceae	Peptoclostridium
6	Firmicutes	Erysipelotrichia	Erysipelotrichales	Erysipelotrichaceae	Erysipelatoclostridium
6	Firmicutes	Negativicutes	Selenomonadales	Veillonellaceae	Veillonella
6	Proteobacteria	Betaproteobacteria	Burkholderiales	Oxalobacteraceae	Ambiguous_taxa
6	Actinobacteria	Actinobacteria	Actinomycetales	Actinomycetaceae	Varibaculum
6	Bacteroidetes	Bacteroidia	Bacteroidales	Prevotellaceae	Prevotella_6
6	Bacteroidetes	Bacteroidia	Bacteroidales	Rikenellaceae	Alistipes
6	Firmicutes	Clostridia	Clostridiales	Family_XI	Ezakiella
6	Firmicutes	Clostridia	Clostridiales	Family_XIII	Mogibacterium
5	Actinobacteria	Coriobacteriia	Coriobacteriales	Coriobacteriaceae	Collinsella
5	Firmicutes	Clostridia	Clostridiales	Peptococcaceae	Peptococcus
5	Firmicutes	Clostridia	Clostridiales	Ruminococcaceae	Ruminococcaceae_UCG_004
5	Firmicutes	Clostridia	Clostridiales	Ruminococcaceae	Ruminococcaceae_UCG_009
5	Firmicutes	Clostridia	Clostridiales	Ruminococcaceae	Subdoligranulum
5	Firmicutes	Erysipelotrichia	Erysipelotrichales	Erysipelotrichaceae	Faecalicoccus
5	Proteobacteria	Epsilonproteobacteria	Campylobacterales	Campylobacteraceae	Campylobacter
5	Bacteroidetes	Bacteroidia	Bacteroidales	Porphyromonadaceae	Dysgonomonas
5	Bacteroidetes	Bacteroidia	Bacteroidales	Porphyromonadaceae	Porphyromonas
5	Firmicutes	Bacilli	Lactobacillales	Streptococcaceae	Streptococcus
5	Actinobacteria	Actinobacteria	Micrococcales	Micrococcaceae	Rothia
5	Firmicutes	Clostridia	Clostridiales	Lachnospiraceae	Eisenbergiella
5	Firmicutes	Clostridia	Clostridiales	Lachnospiraceae	Lachnoclostridium
5	Firmicutes	Clostridia	Clostridiales	Lachnospiraceae	Lachnospiraceae_ND3007_group
4	Firmicutes	Clostridia	Clostridiales	Lachnospiraceae	Roseburia
4	Firmicutes	Clostridia	Clostridiales	Lachnospiraceae	Eubacterium_eligens_group
4	Actinobacteria	Coriobacteriia	Coriobacteriales	Coriobacteriaceae	Atopobium
4	Firmicutes	Clostridia	Clostridiales	Lachnospiraceae	Eubacterium_xylanophilum_group
4	Firmicutes	Clostridia	Clostridiales	Lachnospiraceae	Ruminococcus_gnavus_group
4	Firmicutes	Clostridia	Clostridiales	Lachnospiraceae	Ruminococcus_torques_group
4	Firmicutes	Clostridia	Clostridiales	Ruminococcaceae	Anaerotruncus
4	Firmicutes	Clostridia	Clostridiales	Ruminococcaceae	Ruminococcaceae_NK4A214_group
4	Firmicutes	Clostridia	Clostridiales	Ruminococcaceae	Ruminococcaceae_UCG_005
4	Firmicutes	Erysipelotrichia	Erysipelotrichales	Erysipelotrichaceae	Clostridium_innocuum_group
4	Tenericutes	Mollicutes	Mollicutes_RF9	uncultured_bacterium	uncultured_bacterium
4	Bacteroidetes	Bacteroidia	Bacteroidales	Prevotellaceae	Prevotella_7
4	Firmicutes	Bacilli	Lactobacillales	Carnobacteriaceae	Other
4	Firmicutes	Clostridia	Clostridiales	Family_XI	Anaerococcus
4	Firmicutes	Clostridia	Clostridiales	Family_XI	Finegoldia
4	Firmicutes	Clostridia	Clostridiales	Family_XIII	Family_XIII_UCG_001
4	Firmicutes	Clostridia	Clostridiales	Lachnospiraceae	Anaerostipes
4	Firmicutes	Clostridia	Clostridiales	Lachnospiraceae	Coprococcus_1
4	Firmicutes	Clostridia	Clostridiales	Lachnospiraceae	Coprococcus_2
4	Firmicutes	Clostridia	Clostridiales	Lachnospiraceae	Lachnospira
4	Firmicutes	Clostridia	Clostridiales	Lachnospiraceae	Lachnospiraceae_FCS020_group
4	Firmicutes	Clostridia	Clostridiales	Lachnospiraceae	Lachnospiraceae_UCG_001
3	Firmicutes	Clostridia	Clostridiales	Lachnospiraceae	Lachnospiraceae_UCG_004
3	Firmicutes	Clostridia	Clostridiales	Lachnospiraceae	Eubacterium_fissicatena_group
3	Firmicutes	Clostridia	Clostridiales	Lachnospiraceae	Eubacterium_rectale_group
3	Firmicutes	Clostridia	Clostridiales	Ruminococcaceae	Butyricicoccus
3	Firmicutes	Clostridia	Clostridiales	Ruminococcaceae	Ruminococcaceae_UCG_013
3	Firmicutes	Erysipelotrichia	Erysipelotrichales	Erysipelotrichaceae	Erysipelotrichaceae_UCG_003
3	Fusobacteria	Fusobacteriia	Fusobacteriales	Fusobacteriaceae	Fusobacterium
3	Lentisphaerae	Lentisphaeria	Victivallales	Victivallaceae	Victivallis
3	Proteobacteria	Alphaproteobacteria	Caulobacterales	Caulobacteraceae	Brevundimonas
3	Proteobacteria	Betaproteobacteria	Burkholderiales	Comamonadaceae	Delftia
3	Proteobacteria	Deltaproteobacteria	Desulfovibrionales	Desulfovibrionaceae	Other
3	Actinobacteria	Actinobacteria	Actinomycetales	Actinomycetaceae	Actinomyces
3	Proteobacteria	Gammaproteobacteria	Xanthomonadales	Xanthomonadaceae	Stenotrophomonas
3	Bacteroidetes	Bacteroidia	Bacteroidales	Porphyromonadaceae	Parabacteroides
3	Actinobacteria	Actinobacteria	Bifidobacteriales	Bifidobacteriaceae	Bifidobacterium
3	Bacteroidetes	Flavobacteriia	Flavobacteriales	Flavobacteriaceae	Flavobacterium
3	Firmicutes	Bacilli	Bacillales	Family_XI	Gemella
3	Actinobacteria	Actinobacteria	Corynebacteriales	Corynebacteriaceae	Corynebacterium_1
3	Firmicutes	Clostridia	Clostridiales	Clostridiales_vadinBB60_group	Other
3	Actinobacteria	Actinobacteria	Corynebacteriales	Corynebacteriaceae	Other
3	Firmicutes	Clostridia	Clostridiales	Lachnospiraceae	Dorea
2	Firmicutes	Clostridia	Clostridiales	Lachnospiraceae	Lachnospiraceae_UCG_008
2	Firmicutes	Clostridia	Clostridiales	Lachnospiraceae	Marvinbryantia
2	Firmicutes	Clostridia	Clostridiales	Lachnospiraceae	Tyzzerella_4
2	Firmicutes	Clostridia	Clostridiales	Lachnospiraceae	Ruminococcus_gauvreauii_group
2	Firmicutes	Clostridia	Clostridiales	Lachnospiraceae	uncultured
2	Firmicutes	Clostridia	Clostridiales	Ruminococcaceae	Oscillospira
2	Firmicutes	Clostridia	Clostridiales	Ruminococcaceae	Ruminiclostridium
2	Firmicutes	Clostridia	Clostridiales	Ruminococcaceae	Ruminiclostridium_5
2	Actinobacteria	Coriobacteriia	Coriobacteriales	Coriobacteriaceae	Senegalimassilia
2	Firmicutes	Clostridia	Clostridiales	Ruminococcaceae	Ruminococcus_2
2	Firmicutes	Clostridia	Clostridiales	Ruminococcaceae	Other
2	Actinobacteria	Coriobacteriia	Coriobacteriales	Coriobacteriaceae	uncultured
2	Firmicutes	Erysipelotrichia	Erysipelotrichales	Erysipelotrichaceae	Faecalitalea
2	Firmicutes	Erysipelotrichia	Erysipelotrichales	Erysipelotrichaceae	Turicibacter
2	Proteobacteria	Alphaproteobacteria	Rhizobiales	Brucellaceae	Ochrobactrum
2	Proteobacteria	Betaproteobacteria	Burkholderiales	Alcaligenaceae	Achromobacter
2	Proteobacteria	Betaproteobacteria	Neisseriales	Neisseriaceae	Neisseria
2	Bacteroidetes	Bacteroidia	Bacteroidales	Porphyromonadaceae	Barnesiella
2	Proteobacteria	Gammaproteobacteria	Enterobacteriales	Enterobacteriaceae	Other
2	Proteobacteria	Gammaproteobacteria	Pseudomonadales	Moraxellaceae	Acinetobacter
2	Proteobacteria	Gammaproteobacteria	Other	Other	Other
2	Bacteroidetes	Bacteroidia	Bacteroidales	Porphyromonadaceae	Butyricimonas
2	Tenericutes	Mollicutes	NB1_n	Other	Other
2	Verrucomicrobia	Verrucomicrobiae	Verrucomicrobiales	Verrucomicrobiaceae	Akkermansia
2	Bacteroidetes	Bacteroidia	Bacteroidales	Prevotellaceae	Alloprevotella
2	Bacteroidetes	Bacteroidia	Bacteroidales	Prevotellaceae	Prevotella_9
2	Bacteroidetes	Sphingobacteriia	Sphingobacteriales	Sphingobacteriaceae	Sphingobacterium
2	Firmicutes	Bacilli	Bacillales	Staphylococcaceae	Staphylococcus
2	Firmicutes	Bacilli	Lactobacillales	Enterococcaceae	Enterococcus
2	Firmicutes	Bacilli	Lactobacillales	Enterococcaceae	Other
2	Firmicutes	Bacilli	Lactobacillales	Lactobacillaceae	Lactobacillus
2	Firmicutes	Clostridia	Clostridiales	Clostridiaceae_1	Clostridium_sensu_stricto_1
2	Firmicutes	Clostridia	Clostridiales	Family_XIII	Family_XIII_AD3011_group
2	Firmicutes	Clostridia	Clostridiales	Lachnospiraceae	Lachnospiraceae_NC2004_group
1	Firmicutes	Clostridia	Clostridiales	Lachnospiraceae	Eubacterium_ruminantium_group
1	Firmicutes	Clostridia	Clostridiales	Peptococcaceae	uncultured
1	Firmicutes	Clostridia	Clostridiales	Ruminococcaceae	Oscillibacter
1	Firmicutes	Clostridia	Clostridiales	Ruminococcaceae	Ruminococcaceae_UCG_003
1	Firmicutes	Clostridia	Clostridiales	Ruminococcaceae	Eubacterium_coprostanoligenes_group
1	Firmicutes	Erysipelotrichia	Erysipelotrichales	Erysipelotrichaceae	Holdemanella
1	Firmicutes	Negativicutes	Selenomonadales	Acidaminococcaceae	Acidaminococcus
1	Firmicutes	Negativicutes	Selenomonadales	Acidaminococcaceae	Phascolarctobacterium
1	Firmicutes	Negativicutes	Selenomonadales	Veillonellaceae	Dialister
1	Firmicutes	Negativicutes	Selenomonadales	Veillonellaceae	Megasphaera
1	Proteobacteria	Alphaproteobacteria	Rhizobiales	Brucellaceae	Falsochrobactrum
1	Proteobacteria	Alphaproteobacteria	Rhizobiales	Brucellaceae	Other
1	Proteobacteria	Betaproteobacteria	Burkholderiales	Alcaligenaceae	Alcaligenes
1	Proteobacteria	Betaproteobacteria	Burkholderiales	Alcaligenaceae	Parasutterella
1	Proteobacteria	Betaproteobacteria	Burkholderiales	Comamonadaceae	Comamonas
1	Proteobacteria	Deltaproteobacteria	Desulfovibrionales	Desulfovibrionaceae	Desulfovibrio
1	Proteobacteria	Gammaproteobacteria	Enterobacteriales	Enterobacteriaceae	Ambiguous_taxa
1	Proteobacteria	Gammaproteobacteria	Enterobacteriales	Enterobacteriaceae	Salmonella
1	Proteobacteria	Gammaproteobacteria	Enterobacteriales	Enterobacteriaceae	Tatumella
1	Proteobacteria	Gammaproteobacteria	Pasteurellales	Pasteurellaceae	Other
1	Synergistetes	Synergistia	Synergistales	Synergistaceae	Cloacibacillus
1	Bacteroidetes	Bacteroidia	Bacteroidales	Porphyromonadaceae	Coprobacter
1	Bacteroidetes	Bacteroidia	Bacteroidales	Porphyromonadaceae	Odoribacter
1	Bacteroidetes	Bacteroidia	Bacteroidales	Porphyromonadaceae	uncultured
1	Bacteroidetes	Bacteroidia	Bacteroidales	Prevotellaceae	Prevotella_2
1	Bacteroidetes	Flavobacteriia	Flavobacteriales	Flavobacteriaceae	Chryseobacterium
1	Actinobacteria	Actinobacteria	Corynebacteriales	Corynebacteriaceae	Corynebacterium
1	Firmicutes	Clostridia	Clostridiales	Lachnospiraceae	Butyrivibrio
1	Firmicutes	Clostridia	Clostridiales	Lachnospiraceae	Lachnospiraceae_NK4A136_group
1	Firmicutes	Clostridia	Clostridiales	Lachnospiraceae	Lachnospiraceae_NK4B4_group

**Table A9:** Prevalence of *Bifidobacterium* and *Lactobacillus*

Dataset	Prevalence of *Bifidobacterium* without cut‐off	Prevalence of *Lactobacillus* without cut‐off	Prevalence of *Bifidobacterium* a	Prevalence of *Lactobacillus* a
AGP	79.5%	31.7%	79.5%	31.7%
NBT	100.0%	100.0%	93.1%	39.1%

a Relative abundance of *Bifidobacterium* and *Lactobacillus* over 0.0001.

**Table A10:** The outcomes of the logistic and negative binomial component of the fitted ZINB regression model for *Bifidobacterium*

	Logistic regression component				Negative binomial regression component			
	Estimate	Std. Error	*Z* value	Pr(>|z|)	Estimate	Std. Error	*Z* value	Pr(>|z|)
(Intercept)	−10.841	0.183	−59.086	0	6.284	0.144	43.704	0
Age	0.025	0.002	12.059	0	−0.021	0.001	−17.697	0
Sex								
Female	Reference category							
Male	−0.357	0.067	−5.328	0	0.01	0.042	0.231	0.818
Race								
Caucasian	Reference category							
African American	−0.981	0.553	−1.773	0.076	0.294	0.253	1.163	0.245
Asian or Pacific Islander	−1.116	0.221	−5.061	0	0.734	0.091	8.101	0
Hispanic	−0.603	0.257	−2.344	0.019	−0.251	0.138	−1.821	0.069
Other	−0.087	0.191	−0.454	0.65	−0.113	0.113	−1.005	0.315
Geographic location								
North America	Reference category							
Europe	−0.587	0.074	−7.922	0	0.113	0.047	2.417	0.016
Oceania	−0.02	0.167	−0.122	0.903	−0.338	0.108	−3.141	0.002
Others	0.27	0.507	0.532	0.595	0.996	0.35	2.843	0.004
Whole grain								
Never	Reference category							
Low frequency	−0.471	0.1	−4.709	0	0.271	0.08	3.401	0.001
High frequency	−0.755	0.101	−7.447	0	0.569	0.08	7.119	0
Vegetable								
Never	−0.26	0.342	−0.76	0.447	1.138	0.238	4.773	0
Low frequency	−0.062	0.111	−0.56	0.576	0.221	0.07	3.147	0.002
High frequency	Reference category							
Fruit								
Never	Reference category							
Low frequency	−0.444	0.144	−3.091	0.002	−0.076	0.116	−0.657	0.511
High frequency	−0.69	0.142	−4.851	0	−0.189	0.114	−1.657	0.098
Milk and cheese								
Never	Reference category							
Low frequency	−0.321	0.099	−3.257	0.001	−0.162	0.072	−2.236	0.025
High frequency	−0.374	0.096	−3.907	0	−0.079	0.07	−1.131	0.258
C‐section								
False	Reference category							
True	0.124	0.11	1.132	0.257	0.012	0.067	0.185	0.854
Feeding patterns								
Primarily breast milk	Reference category							
A mixture of breast milk and formula	0.171	0.085	2.01	0.044	0.032	0.056	0.566	0.572
Primarily infant formula	−0.022	0.076	−0.283	0.777	0.077	0.051	1.504	0.133
Antibiotic								
Never	Reference category							
Low frequency	0.251	0.073	3.465	0.001	−0.005	0.048	−0.107	0.915
High frequency	0.11	0.134	0.815	0.415	0.4	0.099	4.021	0
IBD								
False	Reference category							
True	0.133	0.133	1.004	0.315	0.461	0.101	4.558	0
IBS								
False	Reference category							
True	0.264	0.081	3.266	0.001	0.161	0.056	2.887	0.004
Autoimmune disease								
False	Reference category							
True	0.246	0.092	2.68	0.007	−0.15	0.072	−2.1	0.036
Cardiovascular disease								
False	Reference category							
True	−0.179	0.187	−0.955	0.339	0.127	0.128	0.997	0.319
Food allergy								
False	Reference category							
True	0.064	0.07	0.923	0.356	−0.201	0.045	−4.424	0
